# Editorial: Manufacturing, Formulation and Delivery Issues for Phage Therapy to Become A Reality

**DOI:** 10.3389/fmicb.2020.584137

**Published:** 2020-10-08

**Authors:** Danish J. Malik, Gregory Resch

**Affiliations:** ^1^Chemical Engineering Department, Loughborough University, Loughborough, United Kingdom; ^2^Department of Fundamental Microbiology, University of Lausanne, Lausanne, Switzerland

**Keywords:** bacteriophage therapy, quality-by-design (QbD), cGMP Manufacture, upstream process (USP), downstream process (DSP), bacteriophages, antibiotic resistance, formulation/stability

There is resurgent interest in the use of bacteriophages (phages) as medicinal biological therapeutics. This is driven partly by increases in antibiotic resistance in pathogenic bacteria as well as an increasing awareness of the importance of the commensal microbiomes. Indeed, regarding their high species specificity, phages fully respond to the Ehrlich's magic bullet concept (Heynick, 2009), and represent therefore an attractive alternative or adjunct treatment to conventional broad-spectrum antibiotics. Previously concluded phage therapy clinical trials including the Nestlé trial to treat *Escherichia coli* associated diarrhea in children (Sarker et al., 2016) and the PhagoBurn effort by Pherecydes Pharma to treat *Pseudomonas aeruginosa* infections in burn wounds (Jault et al., 2018) however, have highlighted major manufacturing, formulation, and delivery challenges that need to be urgently addressed.

Future personalized medicine approaches may necessitate distributed, near-patient manufacturing of phages. The need to scale-up production to multiple manufacturing sites or nearer to the patient within healthcare settings may require novel manufacturing approaches that are compliant from a regulatory point of view. Flexible modern scalable production approaches following the standards of current Good Manufacturing Practices (cGMP) are needed to supply clinicians with stable phage products of suitable quality, safety, and efficacy. Moreover, personalized medicine implies development of new designs for future randomized clinical trials, which remain mandatory to demonstrate the considerable potential of phage therapy (Bretaudeau et al.).

Increasing manufacturing robustness and standardizing phage production processes under the guidelines of cGMP to guarantee patient safety is the focus of the mini-review by Mutti and Corsini. The authors advocate using a quality-by-design approach to guide phage product and process development at each step. The team responsible for manufacturing phage cocktail batches for the Phagoburn trial share their experiences in a mini-review on cGMP compliance for phage therapy medicinal products (Bretaudeau et al.). Overall, the need for consistent manufacturing processes with a rigorous Quality Assurance (QA) system in place is deemed essential and experience in the field of vaccines for human and veterinary use may be a useful future guide. An important manufacturing consideration relies on the selection of suitable bacterial production strains that preferably should be devoid of virulence determinants and of potential contaminant temperate phages (Philipson et al., 2018). All authors agree that optimization of the upstream (USP) and downstream purification processes (DSP) is crucial to ensure consistent yields and quality. Lastly, consideration of the final formulation and early initiation of stability studies are deemed essential. All-in-one characterization of a multivalent phage cocktail as a medicinal drug product is highlighted as challenging and requiring future development of suitable analytical methods. Development of on-line (real-time measurement within the process) and at-line (sample taken away and measurements taken off-line) metrology techniques for precise enumeration and characterization of the biophysical properties of phage drug products as part of QC testing needs further research.

The purity of phage medicinal drug products is not only an important safety consideration but impacts on phage stability as well. Hietala et al. demonstrate the utility of ultrafiltration for phage concentration as well as buffer exchange for the efficient removal of small bacterial protein toxins. A general low-cost scalable method for removing bacterial endotoxin remains a challenge although encouraging results are presented for a commercial affinity column or using anion exchange chromatography. Accordingly, the paper highlights that the DSP purification stack needs to not only meet relevant purity threshold criteria but also allow high product recovery, which could be challenging and an area for future process development research.

Dry powder formulation of phages is an important Research Topic regarding drug administration through inhalation for patients suffering from lung infection (Malik, 2020). It is also as an appealing way to improve phage storage stability, e.g., in countries where a reliable cold supply chain is a problem (Carrigy et al., 2019; Vinner et al., 2019a). Atmospheric spray freeze drying (ASFD) of phages formulated using trehalose and mannitol as excipients is explored in the original research paper by Ly et al. as an alternative process in preserving phage biopharmaceutical products. ASFD has advantages over conventional lyophilisation methods, such as tray freeze drying, in terms of better heat and mass transfer rates, resulting in considerably faster processing times. There is also better control over the microstructure of the resulting spray dried powders, which can ease re-solubilization prior to administration (Malik et al., 2017).

Developing new approaches for oral administration and improving bioavailability of phages for human therapeutic and animal biocontrol targeting gastrointestinal applications is another important research area (Vinner et al., 2019b). Protecting phages from stomach acidity and increasing the residence time of phages through interaction of encapsulated phages with the intestinal mucosa has previously been shown (Colom et al., 2015). The original research paper by Otero et al. in this special issue explores the use of fluorescently labeled phages encapsulated in liposomes and employs a non-invasive imaging system to track the biodistribution and residence time of the phages *in vivo* in a mouse model.

In summary, this special issue brings together a number of original research articles and mini-reviews that address important bacteriophage manufacturing (including cGMP manufacture) and formulation challenges to ensure future medicinal phage drug products meet quality, safety and efficacy criterion.

## Author Contributions

DM and GR were joint co-editors of this special issue and co-wrote the editorial. Both authors contributed equally to the article and approved the submitted version.

## Conflict of Interest

The authors declare that the research was conducted in the absence of any commercial or financial relationships that could be construed as a potential conflict of interest.
